# Modeling the Brazilian Cerrado land use change highlights the need to account for private property sizes for biodiversity conservation

**DOI:** 10.1038/s41598-024-55207-1

**Published:** 2024-02-24

**Authors:** Carina Barbosa Colman, Angélica Guerra, André Almagro, Fabio de Oliveira Roque, Isabel M. D. Rosa, Geraldo Wilson Fernandes, Paulo Tarso S. Oliveira

**Affiliations:** 1 Faculty of Engineering, Architecture and Urbanism, and Geography, Federal University of Mato Grosso do Sul, CxP 549, Campo Grande, Mato Grosso do Sul 79070-900 Brazil; 2 Programa de Pós-Graduação em Ecologia e Conservação, Universidade Federal de Mato Grosso do Sul, Campo Grande, Brazil; 3 Instituto Terra Brasilis de Desenvolvimento Socioambiental (ITB), Brasília, Brazil; 4 Knowledge Center for Biodiversity —Brazil, Belo Horizonte, MG Brazil; 5 Centre for Tropical Environmental and Sustainability Science and College of Science and Engineering, James Cook University, Cairns, QLD 4811 Australia; 6 School of Natural Sciences, Bangor University, Bangor, Gwynedd LL57 2DG UK; 7 Universidade Federal de Minas Gerais, Belo Horizonte, MG Brazil; 8 Brazilian Knowledge Center on Biodiversity, Belo Horizonte, MG Brazil

**Keywords:** Agrarian structure, Agriculture, Environmental law, Farms, Sustainable ecosystems, Vegetation loss, Conservation biology, Ecosystem services, Forest ecology, Tropical ecology, Environmental sciences

## Abstract

Simulating future land use changes can be an important tool to support decision-making, especially in areas that are experiencing rapid anthropogenic pressure, such as the Cerrado–Brazilian savanna. Here we used a spatially-explicit model to identify the main drivers of native vegetation loss in the Cerrado and then extrapolate this loss for 2050 and 2070. We also analyzed the role of property size in complex Brazilian environmental laws in determining different outcomes of these projections. Our results show that distance to rivers, roads, and cities, agricultural potential, permanent and annual crop agriculture, and cattle led to observed/historical loss of vegetation, while protected areas prevented such loss. Assuming full adoption of the current Forest Code, the Cerrado may lose 26.5 million ha (± 11.8 95% C.I.) of native vegetation by 2050 and 30.6 million ha (± 12.8 95% C.I.) by 2070, and this loss shall occur mainly within large properties. In terms of reconciling conservation and agricultural production, we recommend that public policies focus primarily on large farms, such as protecting 30% of the area of properties larger than 2500 ha, which would avoid a loss of more than 4.1 million hectares of native vegetation, corresponding to 13% of the predicted loss by 2070.

## Introduction

Simulating land use change trajectories considering different legal scenarios has been a powerful approach to decision-making^1^ because it enables us to evaluate the costs and benefits of certain decisions^2^. This is particularly relevant for regions that are undergoing rapid changes such as the biodiversity hotspots on the planet^3^.

The Cerrado hotspot is the largest and most threatened tropical savanna in the world^4^ and has only 52% of native vegetation^5^. The rate of deforestation in the Cerrado has been historically higher than in the Brazilian Amazon, and the expansion of agriculture over the last 30 years was the main driver of these changes^6^. As Brazil is one of the largest producers and exporters of grains and meat (FAO^7^), the Cerrado has become one of the main agricultural areas in the world^8^. This is mainly because of its favorable topographic conditions (flat and smooth undulating relief), soils suitable for agricultural mechanization, and low land prices^6,9^.

In addition to being an important ecological and agricultural region for Brazil, the Cerrado is crucial for the country’s water resource dynamics, as it comprise 10 out of the 12 major Brazilian hydrographic regions^10^. Furthermore, the Cerrado provides ecosystem services of main importance^11^. Sawyer et al.^12^ listed a range of ecosystems services provided by the Cerrado biome, which includes provisioning (e.g. rivers, medications, wood, food, genetic resources, livelihood supplementary income, and hydroelectricity), regulating (e.g. hydrological cycle, storage, and carbon sequestration, and avoided carbon emissions), supporting (e.g. biodiversity, species protection, and pollination), and cultural services (tourism, recreation, sacred lands, and cultural values).

These ecosystem services require high environmental costs for maintenance, owing to fragmentation, biodiversity loss, invasive species, soil erosion and degradation, water pollution, and soil degradation^9^. Despite its importance, the Cerrado has only about 8.3% of formally protected areas, compared to 28% in the Amazon, 9.5% in the Forest Atlantic, 8.8% in the Caatinga, 4.6% in the Pantanal and 3% in the Pampa. About 90% of the biome is privately owned, where a large part of its remaining vegetation is concentrated^13^. The size of properties is a proxy for financial and managerial success, access to information, and compliance with environmental laws. Although some studies have already demonstrated this^14–16^, no study has so far been carried out simulating scenarios explicitly integrating the role of property size in determining future land use changes for the entire territory of the Cerrado.

The Native Vegetation Protection Law (NVPL), popularly known as New Forest Code, is a set of norms that regulate the exploration, conservation, and recovery of native vegetation in Brazil, and it is defined by Law No. 12651 sanctioned in 2012. The NVPL defines the percentage of native vegetation area in a given rural property that must be conserved or preserved, the area that can be converted (for agriculture or cattle), as well as defines situations in which landowners are required to recover natural vegetation. The NVPL brings two legal instruments from the previous Forest Code (Law No. 4771/65) which are of main importance to the natural vegetation conservation, preservation, and recovery: Areas of Permanent Protection (APPs) and Legal Reserves (LRs). The first are protected areas inside the rural property that aims to the preservation water resources, landscape features, geological stability, and biodiversity, facilitate fauna and flora gene flow, conserve soil, and ensure human well-being. The latter are areas inside the rural property with the function of ensuring the sustainable use of natural resources, assisting the conservation and recovery of ecological processes, and promoting the protection of biodiversity. At the same time that the NVPL brought relevant advances that might allow the effective implementation of protection measures to protect and recover native vegetation in Brazil, there are significant setbacks that must be considered, such as the legalization of former illegal deforestation in APPs and the possibility to compensate LRs deficits in other areas covered by native vegetation (within the same biome)^17^. Regardless, compliance with the NVPL is key for the preservation of what is left of the Brazilian flora, fauna, and water resources^18^.

The percentage of the area to be defined as LR depends on the location of the rural property, being 80% of the area in the Amazon biome within the Legal Amazon, 35% of the area in the Cerrado biome within the Legal Amazon, and 20% of the area for the rest of the Brazilian territory. States also have the power to be more restrictive in this amount. The Cerrado is the Brazilian biome with the largest Legal Reserve deficit (minimum percentage of native vegetation required within private properties) and has around 4.2 million ha of native vegetation that needs to be recovered^19^. Furthermore, 40% of its native vegetation can be legally converted^13^. Following the current rate of loss, the ecosystem could disappear by 2030, according to estimates from Conservation International^20^. Reference^13^ showed that by 2050 the Cerrado may lose 40.3 million ha of native vegetation, leaving only 32% of native vegetation. This massive conversion of land use could result in the extinction of about 1140 endemic species by 2050^21^ and perhaps in multiple tipping points, something not evaluated so far for this extraordinary and key biome for world food security and climate regulation.

Studies have shown the importance of assessing land cover and land use change (LCLUC) under multiple scenarios, which can also guide attitudes, choices, and actions that increase the probability of realizing a desirable future^22^. Schaldach et al.^23,24^ and Hampf et al.^25^ also applied an LCLUC model to identify the conversion of vegetation cover to agricultural lands and the consequently increases/decreases in greenhouse gas emissions, providing valuable information to support the development of land use strategies in a portion of the Cerrado biome, in the called Southern Amazon. Some studies evaluate future land use scenarios englobing the Cerrado^26^; however, to the best of our knowledge, there are no studies on scenarios of LCLUC that simultaneously aim to (1) understand which variables influence vegetation loss in the entire Cerrado and whether they change between periods, evaluating (2) which areas are most affected and how much will be lost at a property scale (the management unit of the LR policy). The importance of the Cerrado for both biodiversity and the national economy has led to disagreement among decision-makers, and scientific knowledge is essential to bring a balance to economic development and environmental conservation^27^. Model-based scenarios can be a useful tool in providing information to the decision-making of public and private power^28^ and in reconciling agricultural production and conservation of the Cerrado. Here we used a spatially-explicit model to i) identify the most important drivers of native vegetation loss in the Cerrado; and ii) generate projections of native vegetation loss for 2050 and 2070, considering the trend of recent years and assuming full implementation of the Native Vegetation Protection Law (NVPL), and considering the implications of simulations on the property scale.

## Methods

### Study area

**Figure 1:**
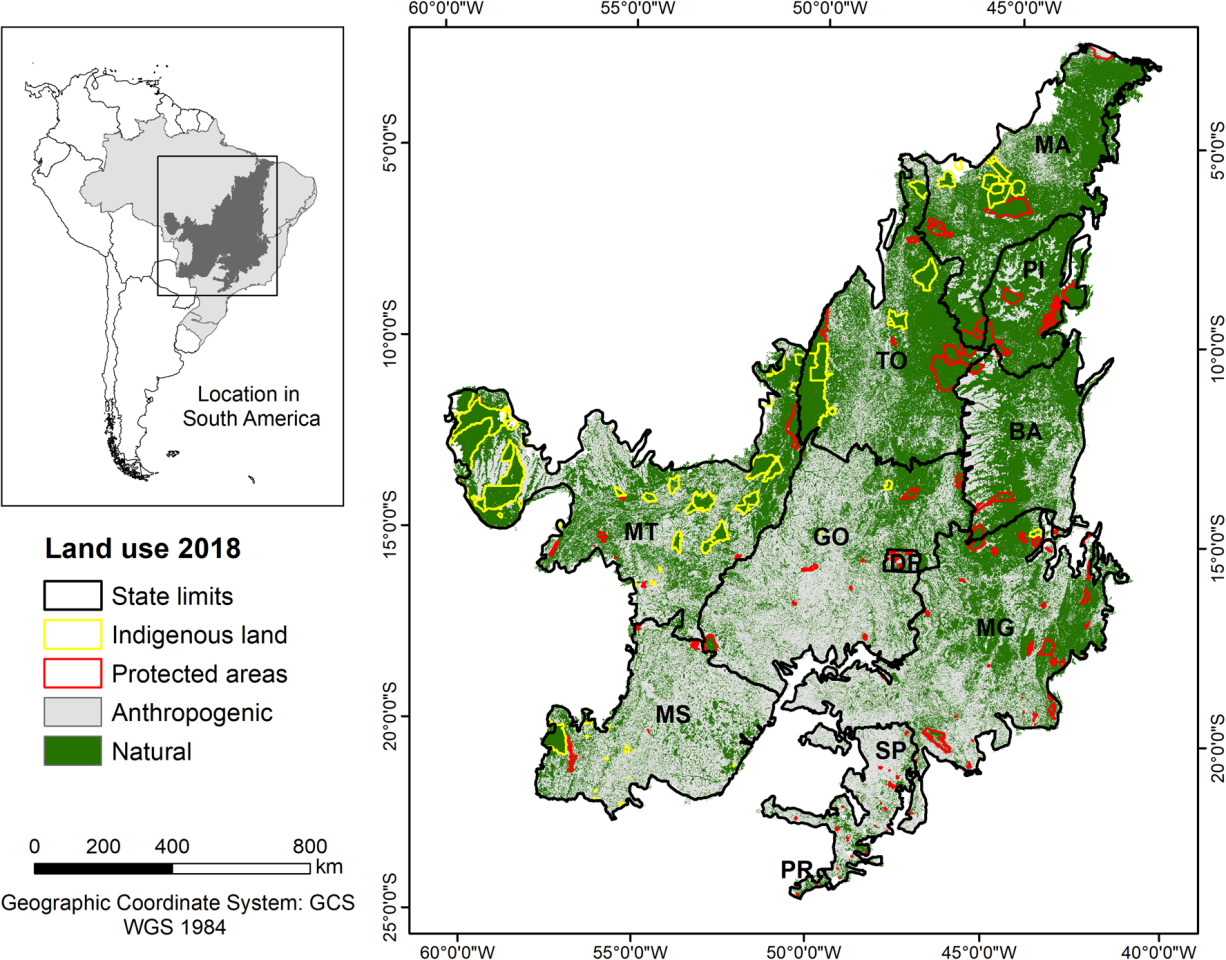
Study area. States included in the Cerrado hotspot: Bahia (BA), Maranhão (MA), Tocantins (TO), Piauí (PI), Mato Grosso do Sul (MS), Mato Grosso (MT), Goiás (GO), Distrito Federal (DF), Minas Gerais (MG), São Paulo (SP), and Paraná (PR). The areas highlighted in yellow are indigenous land and the areas highlighted in red are protected areas.

The Cerrado, also known as the Brazilian Savanna, covers an area of 2 million km^2^ of Brazilian territory (about 24% of the total area), including the Distrito Federal and part of eleven states (Fig. 1). The biome has been classified as one of the 36 global biodiversity hotspots^29^ and it is one of the most important biomes in Brazil, surrounded by four other biomes: the Amazon, Caatinga, Pantanal, and Atlantic Forest.

According to the Köppen climate classification system^30^, the predominant climate groups of the Cerrado are Aw—equatorial, dry winter (83% of the Cerrado); Cwb—dry winter, warm temperate, hot summer (8% of the Cerrado); Cfa—humid, hot temperate, hot summer (5% of the Cerrado); Cwa—dry winter, warm temperate, hot summer (4% of the Cerrado). The average annual rainfall of the Cerrado is approximately 1500 mm, with lower values (close to 700 mm) in the Northeast region, in the transition zone between the Cerrado and Caatinga biomes. The highest average annual precipitation (greater than 2000 mm) is in the Northwest, in the transition area between the Cerrado and the Amazon Forest. The rainy season is from October to March, and the dry season is from April to September^10^.

The predominant soil types, classified according to the Brazilian Soil Classification System (SiBCS) are Latosols (~ 41%), Neossols (~ 23%), Argisols (~ 12%), and Plintosols (~ 10%)^10^. In general, they are weather-resistant and very acidic soils, with little organic matter and nutrients, especially nitrogen and phosphorus^9^. The most common anthropogenic use is pasture (~ 30%), mainly for producing meat and agriculture (~ 9%) with the predominance of annual crops of soybean (90%), cotton (7%), and corn (3%)^31^. The recent expansion of agricultural production occupies approximately 50% (~ 1 million km^2^) of the Cerrado area and in recent years the expansion has occurred mainly towards the northern and more preserved region of the biome, known as MATOPIBA (states of Maranhão, Tocantins, Piauí, and Bahia)^32^.

### Data source

The variables included in the model were identified as possible predictors of Cerrado vegetation loss based on a literature review (Table S1). We used the rural properties of the “Cadastro Ambiental Rural” (CAR; Rural Environmental Registry), and the Legal Reserve (LR) values as a scale for calculating the loss of vegetation according to the Native Vegetation Protection Law—NVPL (Brazil, # 12651, of 2012), which establishes 20% of the legal reserve for Cerrado areas and 35, 50 and 80% for the Legal Amazon (see^13,18^).

There were two types of variables, namely static variables that do not vary within a short time (e.g., distance to roads, cities, and rivers, protected areas, dry season length, elevation, agricultural potential, and property size) (Fig. S1) and dynamic variables, which are those that vary over time (e.g., cattle, permanent and annual crop agriculture) (Fig. S2). All data were converted to the same resolution (1 km × 1 km) and projected onto the same geographic projection (WGS 1984 UTM).

### Land-cover change model

To identify the variables (or drivers) that mainly cause vegetation loss in the Cerrado, we used a spatially-explicit model^33,34^. This model has been used to predict the loss of vegetation at the scale of properties considering different legal requirements and it has already been successfully applied and validated in the Amazon^33^, Pantanal^35^, and Cerrado^36^. The first step of the model involves identifying the drivers that cause vegetation loss in the Cerrado from previous periods. In the second step, the model projects the native vegetation loss for future periods based on the identified drivers.

The model is based on *Pnvl,x,t*, where *Pnvl* is the probability that a ‘native vegetation’ cell *x* is converted into ‘anthropogenic use’ within a defined time interval *t*. The fact that Pnvl,x,t is specific for a given time t illustrates how the model updates the suppression of local native vegetation over time. This probability was defined as a logistic function:

$${\text{P}}_{{{\text{nvl}},{\text{x}},{\text{ t}}}} = { 1 }/ \, ({1 } + {\text{ exp}}^{{ - {\text{k}}_{{{\text{x}},{\text{t}}}} }} ).$$

One can then develop linear models for kx,t as a function of the variables that affect x at time t, and explore the effect of different sets of variables using a model selection procedure (Fig. S5 for all modeling steps).

The model uses Monte Carlo Markov Chains (MCMC) to obtain a posterior probability distribution for each parameter, from which the posterior mean and range of credibility can be extracted, given the model structure and data used for calibration. Binary maps of change are produced (1—native vegetation, 0—anthropogenic) for each time period, which are then integrated based on the 100 iterations of the model (sampling from the posterior distributions) to determine the overall probability of change (i.e., if a pixel is selected to be converted 100 times out of 100 iterations it has a 100% probability of conversion in time t). These steps were repeated for each of the four time periods as the model will project future conversion based on observed rates of change, and the periods (2008–2010, 2010–2012, 2012–2014, and 2014–2016) had different rates of change. Once all models were calibrated, the best one (with the combination of variables that yield the highest test likelihood in each calibration time period) was used to project future probabilities of native vegetation loss until 2050 (using two-year time steps). The accumulated probability of conversion by 2050 was determined for each model individually (2008–2010, 2010–2012, 2012–2014, and 2014–2016 models) as well as based on an ensemble of all model outputs (i.e. integrating all model projections made for a particular year). To assess the goodness-of-fit of the models, we calculated the area under the receiver operating characteristic curve (or AUC) values for each period of each analyzed area (Table S2).

We then calibrated the model for four time periods (2008–2010, 2010–2012, 2012–2014, and 2014–2016) attributed to different rates of vegetation loss (Fig. S3), thus leading to potential differences in projected rates (that can be derived from the model). After that, we performed a model ensemble by averaging the projections from the four periods, obtaining the rate of vegetation loss every two years from 2016 to 2070 for the BAU (Business as usual) scenario, which considers the Legal Reserve (LR) amount provided for the properties in NVPL and the current Protected Areas (PA). To assess the goodness-of-fit of the models, we computed the area under the receiver operating characteristic (or AUC) values for each period of each analyzed area (Table S2).

Other scenarios were used in previous studies (see Colman et al.^36^), where we simulated an increase/decrease in the area of LRs and PAs regulated by the NVPL. The trend in native vegetation loss in the four previous periods was considered in all cases.

### Rural properties registered in the Brazilian Cerrado

**Figure 2:**
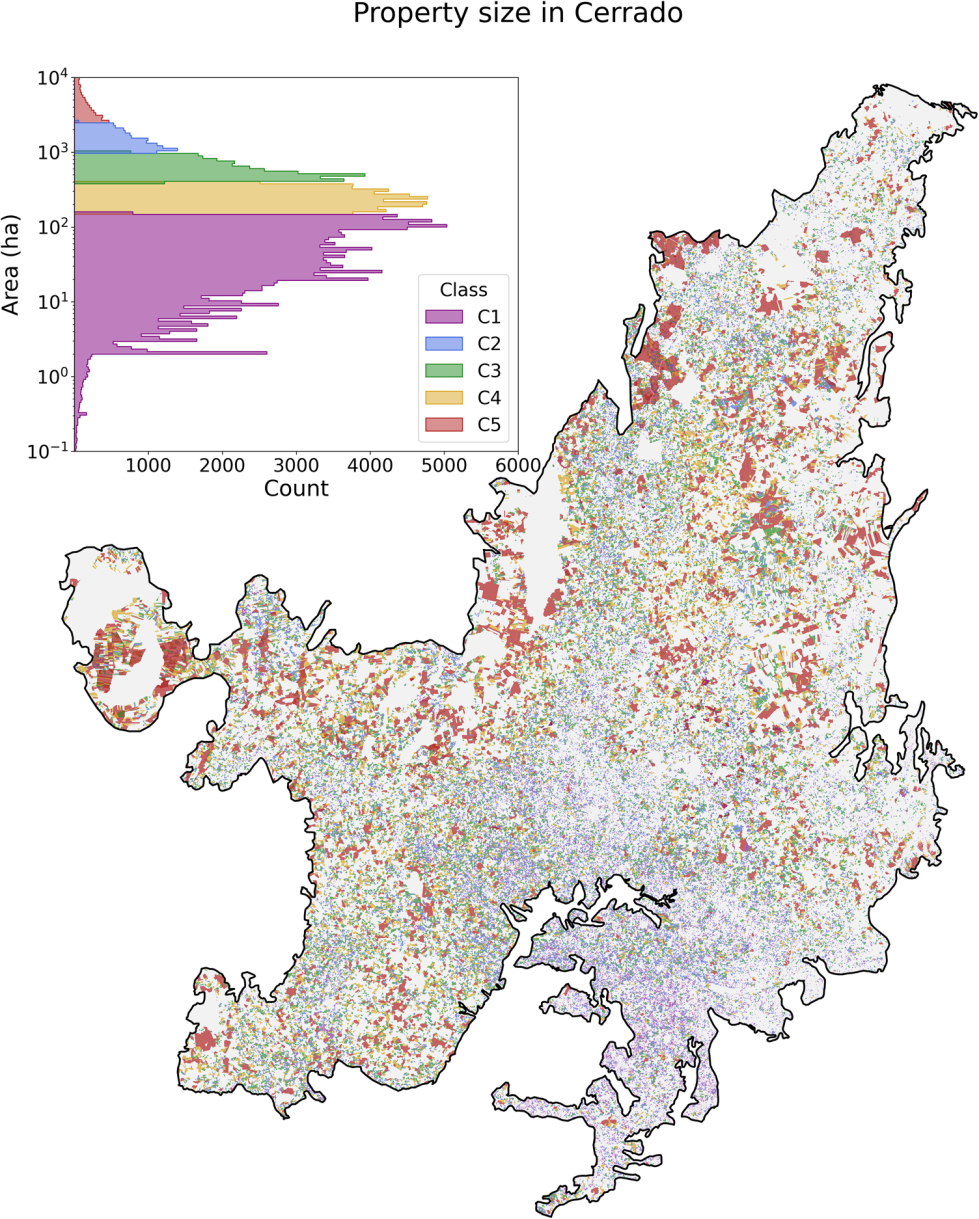
252,007 private rural properties in the Cerrado biome considered in our study. The georeferenced limits of the properties are from the CAR database. The histogram shows the distribution of the rural properties in the classes based on the size of the property. C1 (1 ≤ 150 ha), C2 (150 ≤ 400 ha), C3 (400 ≤ 1000 ha), C4 (1000 ≤ 2500 ha) and C5 (> 2500 ha).

We used the January 2020 CAR database, which had 892,127 properties registered in the Cerrado. Of those registered properties, 252,007 had their georeferenced limits available for download and are on those that our analyses were run. To assess how much vegetation will be lost in small, medium, and large properties we used the classification by Michalski et al.^14^ that considers five classes: C1 (1 ≤ 150 ha), C2 (150 ≤ 400 ha), C3 (400 ≤ 1000 ha), C4 (1000 ≤ 2500 ha) and C5 (> 2500 ha). The classification is also adopted by Stefanes et al.^16^ in the Cerrado of Mato Grosso do Sul. We consider C1 as small properties, C2 and C3 as medium, and C4 and C5 as large properties. Figure 2 shows the spatial distribution of the private rural properties in the Cerrado biome and their distribution within the classes (C1, C2, C3, C4, and C5). Following the above-mentioned, of the 252,007 rural property limits collected, 61% are small, 33% are medium-sized, and 5% are large. The area of large properties covers 48.5% of the area of all collected properties^37^.

## Results

### The main drivers of native vegetation loss in the Cerrado

The variables identified as important to explain the loss of vegetation in the Cerrado were different between the periods analyzed (2008–2010, 2010–2012, 2012–2014, and 2014–2016). Protected areas (including Indigenous lands) indicate a positive impact in all periods, showing a lower probability of native vegetation loss inside these areas. The distance to rivers explained the vegetation loss in three periods (2008–2010, 2010–2012, and 2012–2014), while the distance to cities explained only two periods (2008–2010 and 2014–2016), and the distance to roads only explained 2010–2012 (Table 1). In all periods analyzed, the greater distance from rivers led to a greater loss of native vegetation while the opposite occurred for roads and cities.

**Table 1:** Median of relative contributions of the variables to the final model parameters.

Variables	2008–2010	2010–2012	2012–2014	2014–2016
Land cover	3.773386	2.706573	2.450408	2.547661
Distance to roads	0	− 0.000004	0	0
Distance to cities	0.000009	0	0	− 0.000008
Dry season length	0	0	0	0
Elevation	0	0	0	0
Agricultural potential	0.000225	− 0.000058	0	0
Distance to rivers	0.000078	0.000079	0.00003	0
Cattle	0	0	0.001643	0
Permanent agriculture	0	0	0.000001	0
Annual crop agriculture	0	0.000022	− 8.1E-05	0
Protected areas	− 1.727708	− 1.379807	− 1.45011	− 1.078604

Agriculture and cattle explained native vegetation loss in only one or two periods, whereby the agricultural potential influenced the vegetation loss in 2008–2010 and 2010–2012, and the annual crop agriculture influenced the loss in 2010–2012 and 2012–2014. Permanent agriculture and cattle explained the loss of vegetation in only one period (2012–2014). On the other hand, dry season length and elevation did not explain the loss of vegetation in the Cerrado in any of the periods observed (Table 1).

### Projections native vegetation loss in the Cerrado

According to our projections, the Cerrado may lose 26.1% (± 11.6% 95% C.I.) of the area of native vegetation according to the Legal Reserve limits (excluding protected areas and indigenous lands) by 2050, and 30.2% (± 12.6% 95% C.I.) by 2070. This corresponds to 26.5 million ha (± 11.8 95% C.I.) loss of native vegetation by 2050 and 30.6 million ha (± 12.8 95% C.I.) by 2070. The conversion values of native vegetation varied between the periods analyzed, in which 2008–2010 showed the lowest loss while 2012–2014 had the highest loss (Fig. S4).

**Figure 3:**
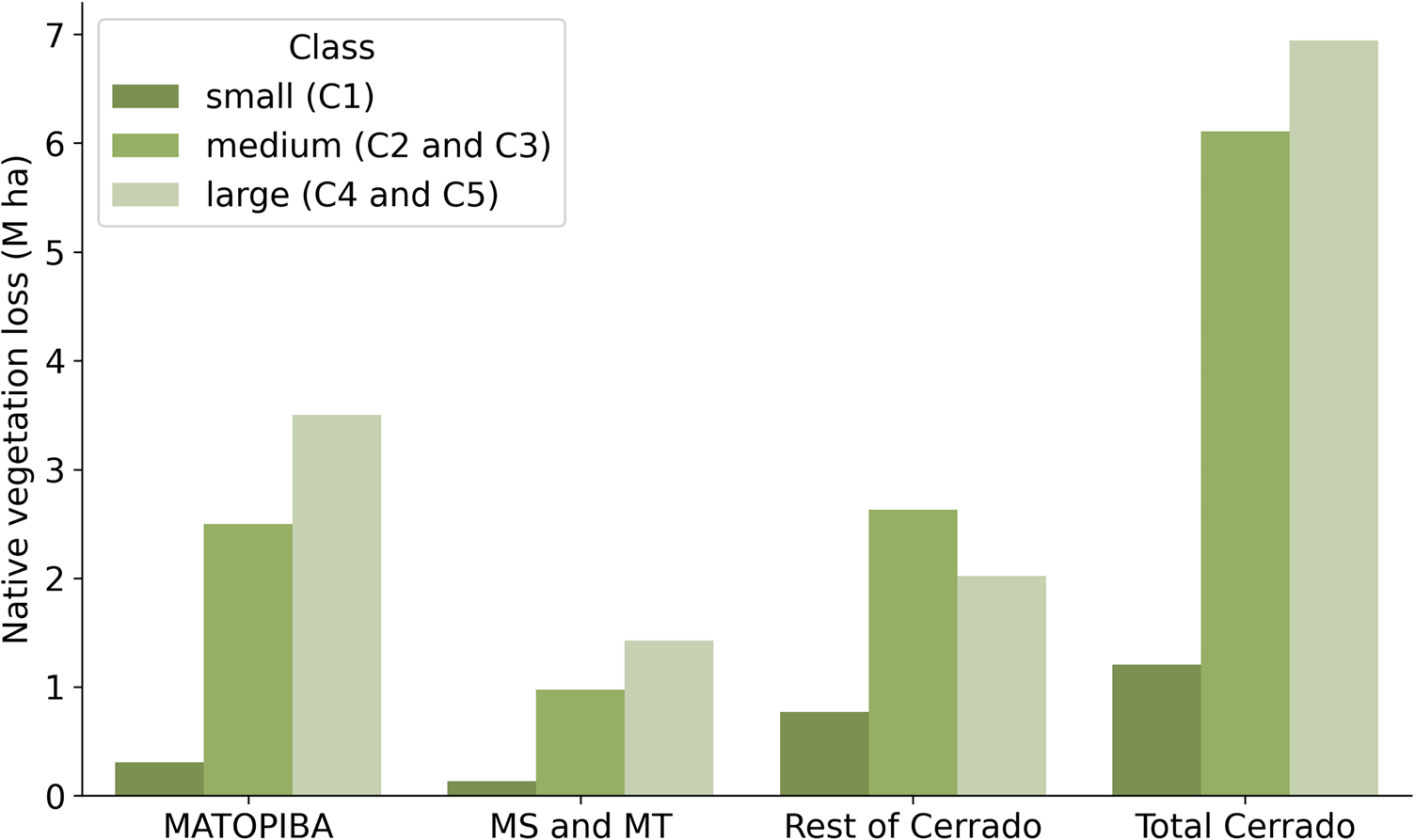
Native vegetation loss per property size in the Cerrado by 2070.

The loss of vegetation in the Cerrado by 2070 will occur mainly in large properties (C4 and C5), adding up to more than 7 million hectares, especially in the MATOPIBA region (Fig. 3). For states that do not include MATOPIBA and Mato Grosso do Sul and Mato Grosso, vegetation loss will occur mainly in medium-sized properties (between 150 and 1000 ha—C2 and C3).

**Figure 4:**
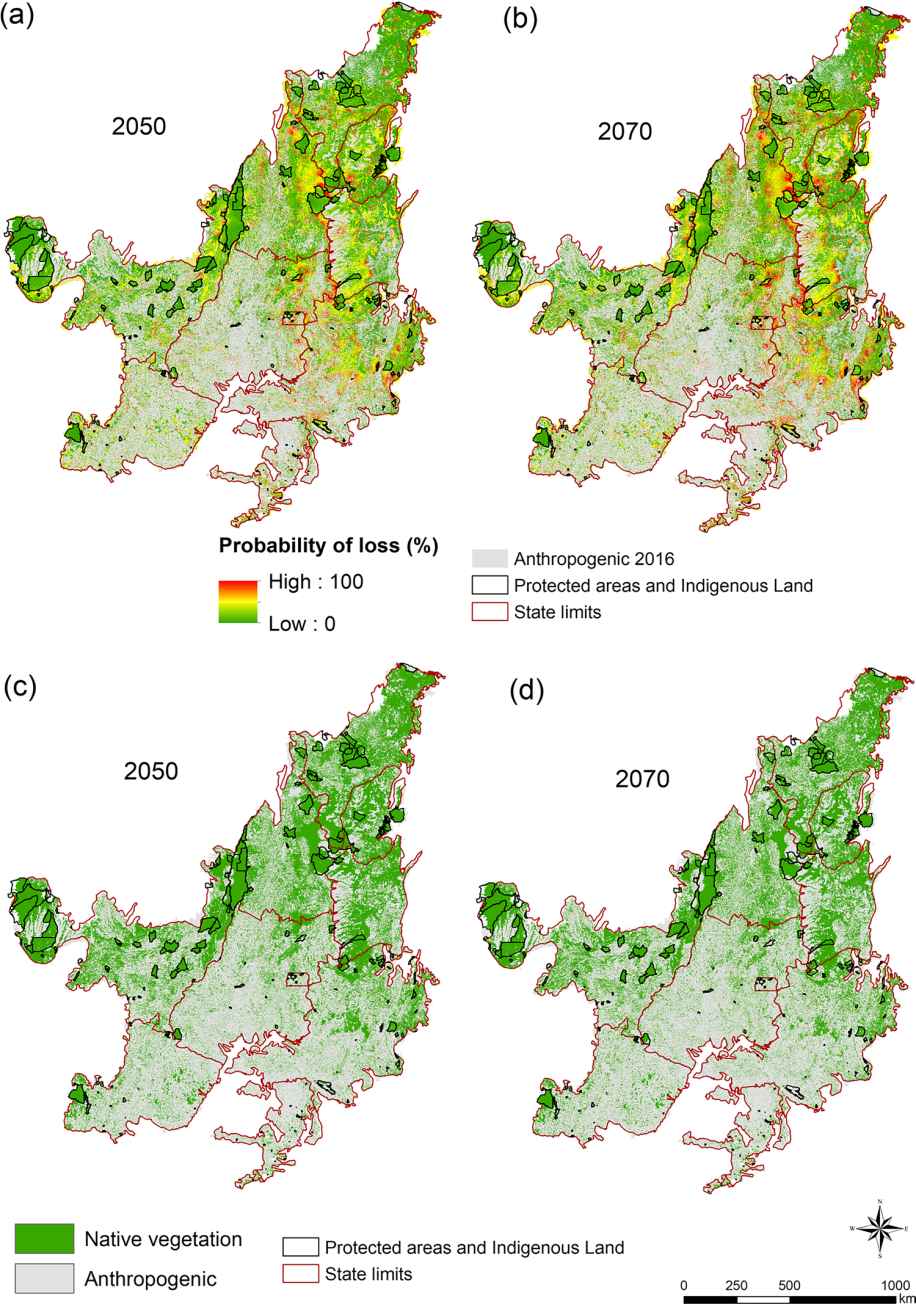
Projections of accumulated native vegetation loss by (**a**) 2050 and (**b**) 2070, and native vegetation remaining for (**c**) 2050 and (**d**) 2070 for the mean values of the four periods (2008–2010, 2010–2012, 2012–2014 and 2014–2016). Protected areas only consider strict protection areas.

We spatialized the projected native vegetation losses in the Cerrado for 2050 and 2070 (Fig. 4a,b). The states with the greatest expected vegetation loss by 2070 are Minas Gerais (22.0%), Tocantins (18.0%), Goiás (14.6%), Mato Grosso (10.6%), and Maranhão (10.4%) (Fig. 4b,d). We generated an animation showing the evolution of the probability of loss of vegetation from 2016 to 2070 (available at http://bit.ly/38u2zl2).

It is clear to note that the evolution of the native vegetation losses is mainly correlated with the spatial distribution of the drivers (Fig. S1). In the state of Minas Gerais, in southern Cerrado, the high agricultural potential, proximity to rivers and roads (which are important to irrigation and transportation), along with a very few numbers of protected areas, and the lowest percentage of area required for Legal Reserves, are driving that region to accumulate about 22% of the expected deforestation. In the northern portion, we expect deforestation hotspots in the MATOPIBA region, led by the high agricultural potential of the region, which is a function of a short dry season, and topography and soils suitable for mechanized agriculture implementation. Our results also indicate that Protected Areas in the MATOPIBA region may be pressured by surrounding deforestation, which will require strong mechanisms, instruments, and surveillance to keep these areas effectively protected.

## Discussion

Our study adds more evidence that under the existing environmental protection framework, the Cerrado hotspot will face rapid land use changes in the coming years if nothing is done to change the current trajectory^1,13,20,21,38^. Our spatial model enabled us to identify areas most likely to lose native vegetation. Moreover, we showed that considering the agrarian structure (the distribution of assets and rights linked to land among populations that live in rural areas or derive a significant income from rural activities^39^), the size of the properties and their probability of land use change could be a very useful tool to support sustainable management plans.

The agrarian structure is very relevant to predicting future trajectories of land use as many decisions are made at this level. In addition, the size of the property is a proxy for political influence. In the Cerrado, small properties are predominant in terms of numbers and large properties in terms of area (these occupy more than 60% of the biome’s area). Furthermore, large properties have a greater tendency to have greater coverage of native vegetation and comply with NVPL, although this relationship was found to be very weak, particularly in the Cerrado of Mato Grosso do Sul^16^. Multiple reasons can explain these patterns: commodity and export markets are highlighted as they can be found in these large properties that seek to meet the minimum requirements determined by the NVPL. Large landowners receive more subsidies from government programs^40^, while smallholders tend to keep less native vegetation on their properties to compensate for the low profitability of their properties^14^. Moreover, this may be a result of the size of the area vs recent activity time.

### The main drivers of native vegetation loss in the Cerrado

Drivers of agricultural dynamics in Brazilian states are characterized by complex spatial and temporal interactions between local, regional, and global influences on land-use and land-cover change within the Cerrado^41^. In our study, the impact of agriculture and livestock on the loss of native vegetation did not unfold as expected, as they accounted for the loss only in some periods. This divergence from expectations may stem from the introduction of agriculture and pasture areas into regions already deforested prior to technological advancements that increased productivity. Alternatively, there might be a time lag between deforestation and the intensification of land use through tree-crop-pasture systems or crop-pasture systems. The technological advancement in commodity production in the Cerrado, largely driven by research breakthroughs from the Brazilian Agricultural Research Corporation (Embrapa) in the 70’s decade, enabled a strong process of intensification and scalability in agricultural production in the region^42^.

The consequences of agricultural intensification on landscape changes, particularly deforestation, are a contentious issue. On one hand, the intensification of agriculture can serve as a safeguard against Cerrado degradation by justifying increased production per unit area, thereby reducing the pressure for new land conversions^41^. However, on the flip side, agricultural intensification can lead to environmental impacts, such as increased risks of over-fertilization of the soils and environmental contamination^43^, and may paradoxically contribute to further deforestation. As productivity per hectare rises, producers might be incentivized to expand their productive areas to enhance earnings. In this context, it is crucial to implement public policies that decouple commodity production from deforestation rates, focusing on evidence-based strategies, incentives, and efforts on managing and intensifying previously cleared lands^41^.

Research conducted in the Cerrado has revealed that a portion of the areas operates under the Integration-Harvest-Livestock-Forest regime, where agriculture expands primarily in pasture areas^44^. This integration involves fields used interchangeably for agriculture and livestock, with tree threads planted between the fields to facilitate cattle foraging. The goal is to intensify land use, encourage crop rotation, and sustain livestock without resorting to native vegetation clearance^42,45^. Importantly, agriculture within the biome still has significant growth potential without jeopardizing preserved areas, as approximately 50 million hectares of underutilized pasture land could be repurposed for agricultural production^1^.

The development of extensive road networks in the Cerrado commenced with the establishment of Brasília in 1956, leading to significant economic growth. However, the construction of these roads had subsequent impacts on native vegetation. In 2009, Brazil invested 0.35% of its GDP (2.2 billion dollars) in highways^46^, and this investment played a role in the loss of vegetation in the Cerrado from 2010 to 2012. Moreover, road construction transformed villages into cities, resulting in increased populations and subsequent loss of native vegetation. While cities have expanded to accommodate population growth, the founding of entirely new cities has been infrequent.

Conversely, the proximity of rivers acted as a protective factor against vegetation loss from 2008 to 2014, highlighting the significance of Areas of Permanent Preservation (APPs) as a legal instrument for safeguarding native vegetation. Moreover, protected areas emerged as crucial in preventing vegetation loss across all analyzed periods. This is evident in Figs. 1 and 4, illustrating that these areas harbor substantial native vegetation amid anthropogenically influenced surroundings in unprotected zones.

Spatial variations in the duration of the dry season and altitude within the Cerrado did not provide an explanatory framework for vegetation loss in the studied periods. This suggests that neither elevation nor drought constrains the expansion of human activities such as agriculture. Notably, the MATOPIBA region, the agricultural frontier of the Cerrado characterized by a prolonged dry season, continues to sustain despite these challenges^8^. Additionally, the development of new technologies and the selection of crop varieties have further facilitated agriculture's expansion into areas that were previously not conducive^42,47^.

### Projections native vegetation loss in the Cerrado

Assuming full implementation of NVPL and continuing the socio-economic trends of the past, the native vegetation in the Cerrado may decrease from 52.0 to 38.7% in 2050 and 36.6% in 2070. Our projections are not as drastic as those from Machado et al.^20^ but more in line with those from Soares-Filho et al.^13^, although slightly higher, possibly due to the recent increase in conversion rates^48^. Although we were able to analyze temporal variation in the drivers of change (covering 12 years), the study does not capture the whole expansion process in the Cerrado that started in the 1950s. For this reason, some variables that seemed weak over the last 12 years may have been key in the past, such as roads and cities. For an overview of the process, it would be important to expand the analysis to the 1950s until now, which unfortunately is not possible due to the lack of data.

The areas with the highest probability of loss occur mainly in Minas Gerais, Goiás, Mato Grosso, and Maranhão. These four states were part of a federal program in 1975 aiming to accelerate economic development through various types of financing, aimed at building roads, silos, warehouses, and agricultural research. Currently, the region is responsible for about 60% of the country’s grain production^8^. MG and GO present most of their area with a requirement of only 20% of legal reserve, although NVPL requires values of legal reserve of 35% and 80% for most of the area of the states of TO and MA. These states are located in MATOPIBA, which is known as the agricultural frontier of the Cerrado, mainly with soy expansion^8^. In addition, the native vegetation is concentrated in the northeastern region of the Cerrado, where large properties with the largest fragments of native vegetation that are susceptible to suppression are found. Therefore, legal instruments or economic incentives for conservation need to be created (e.g. payments for environmental services, and biodiversity credits) for owners to avoid converting surplus native vegetation within consolidated farms, as well as promote the recovery of environmental liabilities^49^. In addition to the incentives, the expansion of the soy moratorium (a zero-deforestation agreement between civil society, industry, and the government that prohibits the purchase of soy grown on recently deforested land in the Brazilian Amazon) is a way to prevent converting areas for purposes of agricultural expansion^38^.

The Native Vegetation Protection Law brought some important advances to the implementation of effective administrative control of deforestation in Brazil. However, the Cerrado biome covers approximately 2,000,000 km^2^ and there are more than 800,000 registered private rural properties in the CAR database. Even with the recent advances in remote sensing techniques and technologies to monitor and identify deforestation (legal and illegal), such as the Deforestation Detection in Real-Time (DETER) and the Amazon Deforestation Satellite Monitoring (PRODES), the lack of surveillance personnel in situ makes it difficult to ensure that all deforestation will occur respecting the Law. At the same time, some regulations of the NVPL are underway in the Brazilian states, which englobes complementary laws, decrees, and resolutions to establish rules and clarify how the Federal Law is to be employed in each state. This can be an opportunity to correct the Federal Law setback but also create a time gap for illegal deforestation.

Our results show evidence that applying NVPL alone is not sufficient for the conservation of the Cerrado, as large areas especially within large properties can be deforested under the protection of the law, explicating that the conversion of native vegetation is in some way facilitated by the weakness of political-administrative control. In this context, there is first a need for inspection so that properties that do not comply with NVPL offset their liabilities. For properties within the law, there is a need to develop actions beyond the existing policies. These policies should focus on keeping the LR rates well above the NVPL and preventing the conversion of natural vegetation. This can be achieved through various measures, including investing in environmental services, enhancing pasture productivity, promoting conservation through economic and market-driven mechanisms (such as carbon and biodiversity credits), incentivizing the expansion of agricultural land on already converted areas, and extending the scope of initiatives like the Soy Moratorium (currently applicable only to the Amazon) to encompass other commodities like sugarcane and beef in native pastures^21^. In addition, incentives must be designed according to the different realities faced by small and large owners, making the actions more profitable and increasing the probability of success^16^. Our study shows that these actions are urgent, especially in the MATOPIBA region, in the agricultural expansion area of the Cerrado, and where there are the largest remnants of native vegetation. More than 70% of soy and about 20% of beef produced in the country are sold on the foreign market, therefore the cattle and soy export chains are fundamental in changing part of the trajectory. Controlling the export chain is a relatively important mechanism for large companies focused on the foreign market^8,32^.

In identifying priority actions within the agrarian structure, in an ideal scenario without political, social, financial, practical, or personal constraints, our recommendation to decision-makers would be to encompass all analyzed properties in an extensive conservation strategy. This strategy would incorporate diverse actions, aligning with proposals articulated by Strassburg et al.^21^. However, we acknowledge the impracticality of such a comprehensive approach due to constraints such as limited time, financial resources, and the intricate web of political, social, and economic considerations. Given these constraints, our results underscore the importance of a targeted approach. Specifically, focusing on selected properties based on size and the likelihood of land conversion in the near future becomes crucial. This strategic focus allows for the development of impactful conservation strategies on a landscape scale, optimizing time, financial resources, and social mobilization efforts. In line with this perspective, we propose an initial emphasis on negotiations with a subgroup of landowners who wield a substantial influence on vegetation loss in the Cerrado. For instance, by encouraging all properties (particularly those in regions requiring 20% to 35% Legal Reserves) with over 2,500 hectares to designate 30% of their areas as protected zones or under sustainable management—complementing the Legal Reserve—significant conservation gains could be achieved. This approach, advocated by some authors to avert abrupt declines in tropical biological diversity, could safeguard over 4.1 million hectares. Notably, this corresponds to 15% of the predicted loss of native vegetation by 2050 and 13% by 2070 in our model.

In terms of harmonizing conservation and agricultural production, a strategic focus on large farms emerges as pivotal. These farms, typically characterized by highly capitalized large-scale commodities and export-oriented production, particularly in regions like MATOPIBA, appear strategic due to their financial robustness. Moreover, they receive substantial incentives from the Brazilian government^50^ and, potentially, possess greater adaptive capacity in the face of climate change and the multifaceted social, economic, and environmental challenges compared to family farmers. Initiating practical efforts with a focus on large properties is also crucial for the conservation of endangered vertebrate species in the Cerrado, as recently underscored by Ref.^51^.

Finally, it is important to make clear that our results are projections generated by a stochastic model. Therefore, there are some uncertainties that need to be recognized here, such as uncertainties related to the data input and model assumptions. For instance, we have used static variables that do not vary within time (e.g., distance to roads, cities and rivers, protected areas, dry season length, elevation, agricultural potential, and property size) (Fig. S1). The lack of updates to these parameters throughout the simulations implies that the influence of variables, such as distance to roads or the effectiveness of protected areas, remains consistent over time. These static variables represent a challenge, as the assumption of unchanging processes in LCLUC may introduce errors in model predictions^33,34^. Additionally, the rural production datasets used in our study came from the national census obtained at the municipal scale (Table S1). Thus, the spatial scale of the datasets used in this study is also a source of uncertainty. Another limitation found in our study is that we used the CAR database provided by January 2020. This dataset does not cover all private properties in the Brazilian Cerrado, and as is a self-declared dataset may have some bias in the properties’ boundaries. However, these limitations do not compromise our main findings, despite that we suggest being considered in future studies on LCLUC in the Brazilian Cerrado.

## Conclusion

In this study, we used a dynamic and spatially-explicit land use change model to identify the main drivers of native vegetation loss in the Cerrado biome. Since the drivers were identified, we projected the estimated native vegetation loss for 2050 and 2070 years. In addition, we analyzed the role of property size in determining different outcomes of these projections. Our results show that distance to rivers, roads, and cities, agricultural potential, permanent and annual crop agriculture, and cattle led to the historical vegetation loss, while protected areas prevented it.

Considering that the current Forest Code is fully adopted, it is projected that the Cerrado could experience a reduction of 26.5 million hectares (± 11.8 95% C.I.) of native vegetation by 2050 and 30.6 million hectares (± 12.8 95% C.I.) by 2070. This deforestation corresponds to ~ 13% and ~ 15% of the Cerrado area by 2050 and 2070, respectively. This may occur mainly in large properties and would be devastating for the Cerrado, leading to further species loss and ecosystem degradation and unknown spillover effects on climate change. Hence, to provide a balance between conservation and agricultural production, we recommend that public policies prioritize large farms. For instance, protecting 30% of the area of properties larger than 2500 ha would avoid a loss of more than 4.1 million hectares of native vegetation, corresponding to 13% of the predicted loss by 2070.

### Supplementary Information

Supplementary Information.

## Data Availability

The datasets generated during the current study are available from the corresponding author on reasonable request.
