# Supplementary material for: Modeling the Brazilian Cerrado land use change highlights the need to account for private property sizes for biodiversity conservation
Source: Sci Rep. 2024 Feb 24;14:4559. doi: 10.1038/s41598-024-55207-1 (PMC10894285; doi:10.1038/s41598-024-55207-1)
Supplement: Supplementary file 1 — Supplementary Information. [file 41598_2024_55207_MOESM1_ESM.doc]

**Supplementary Material**

**Modeling the Brazilian Cerrado land use change highlights the need to account for private property sizes for biodiversity conservation**

Carina Barbosa Colman, Angélica Guerra, André Almagro, Fabio de Oliveira Roque, Isabel M.D. Rosa, Paulo Tarso Sanches de Oliveira^*^

*Corresponding author: paulotarsoms@gmail.com

This file contains:

Supplementary Figure S1

Supplementary Figure S2

Supplementary Figure S3

Supplementary Figure S4

Supplementary Figure S5

Supplementary Table S1

Supplementary Table S2


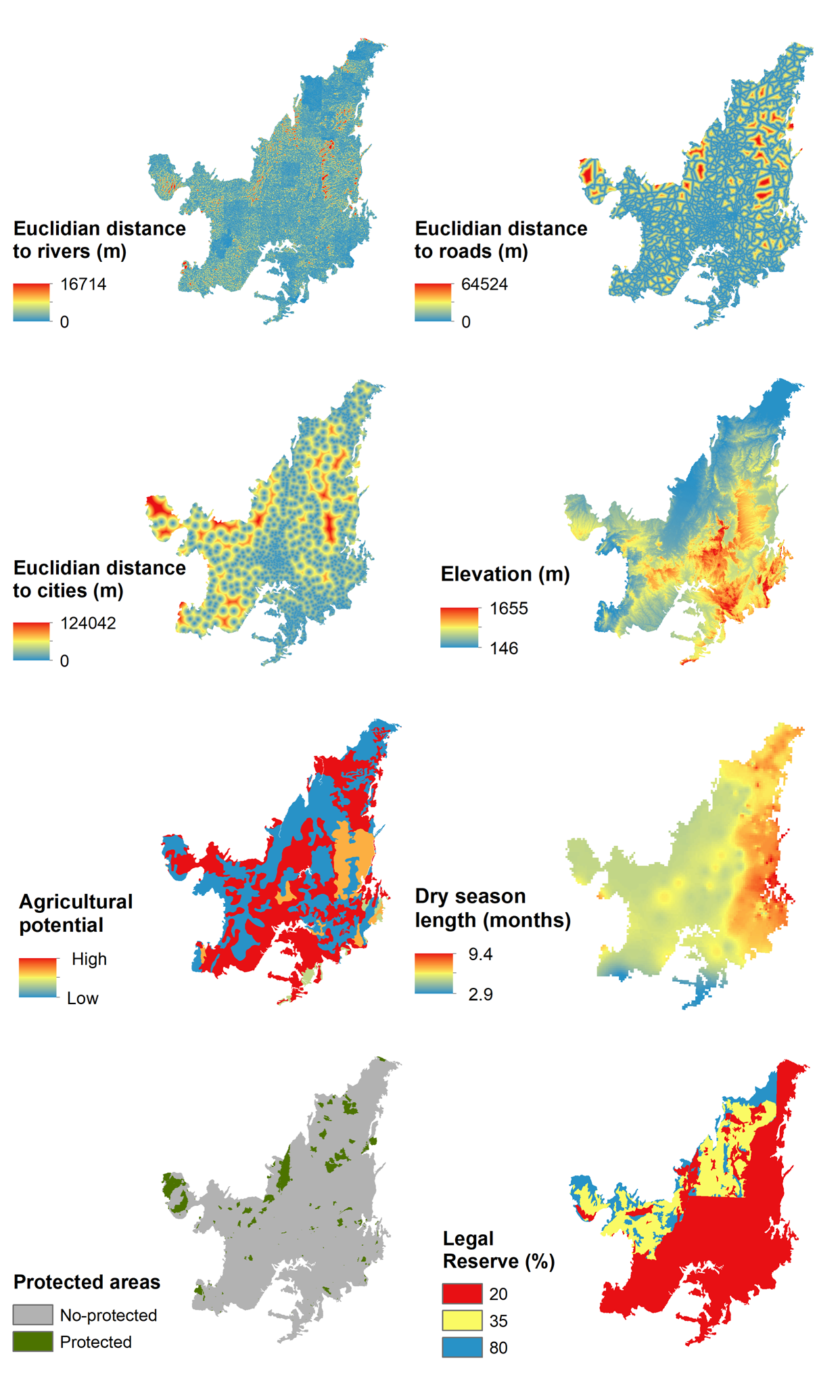


**Figure S1.** Spatialization of the static variables included in the model and the Legal Reserve.


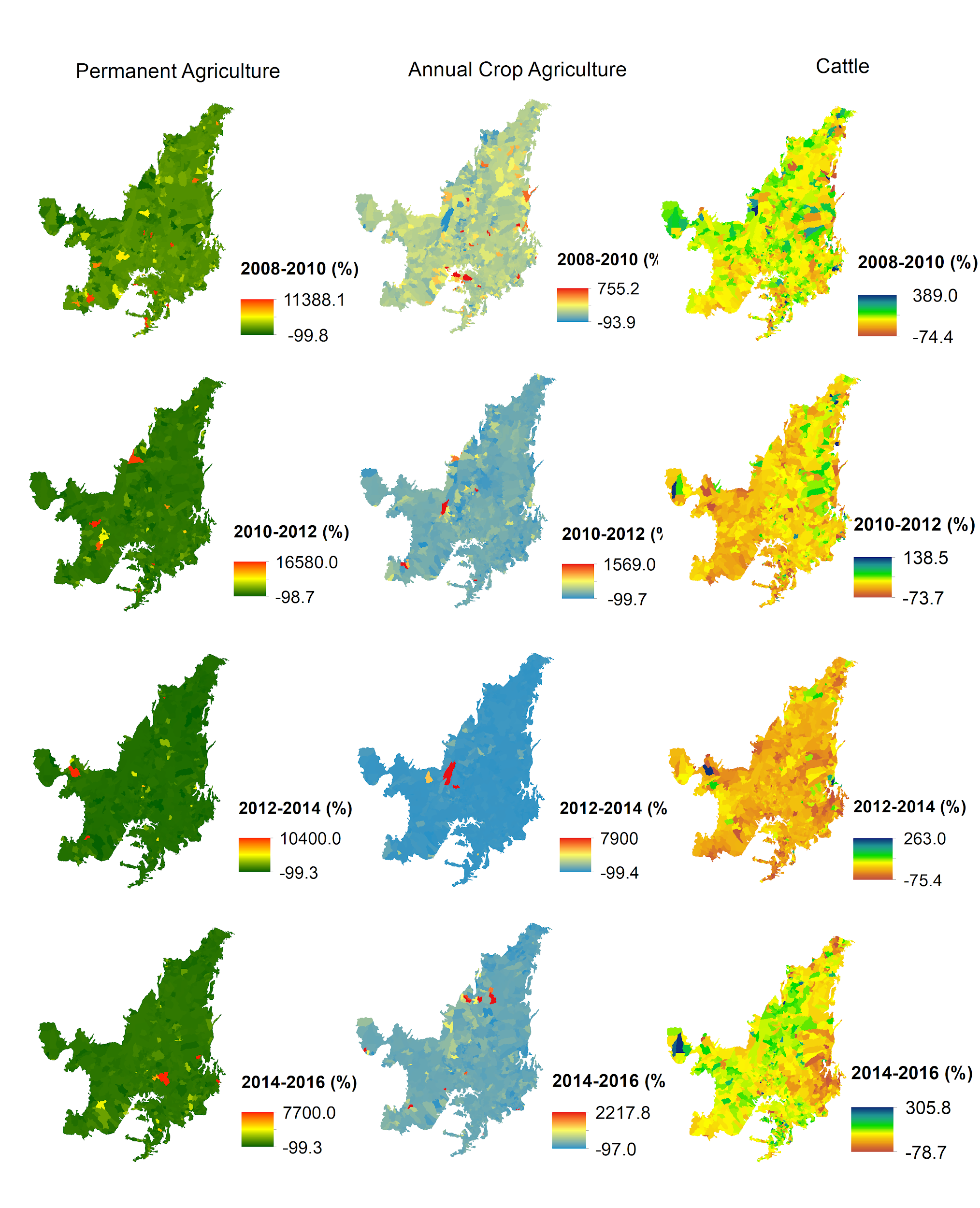


**Figure S2.** Spatialization of the dynamic variables included in the model in the four periods (2008-2010, 2010-2012, 2012-2014 and 2014-2016). Note: the % is the percentage change between t1 and t2.


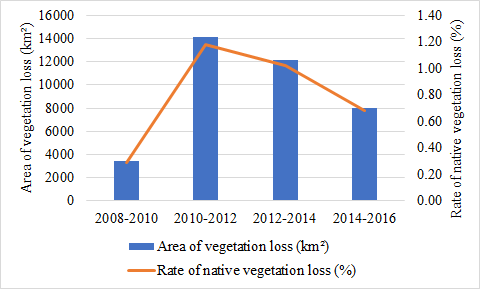


**Figure S3.** Area and rate of native vegetation loss in the Cerrado in the periods analyzed.


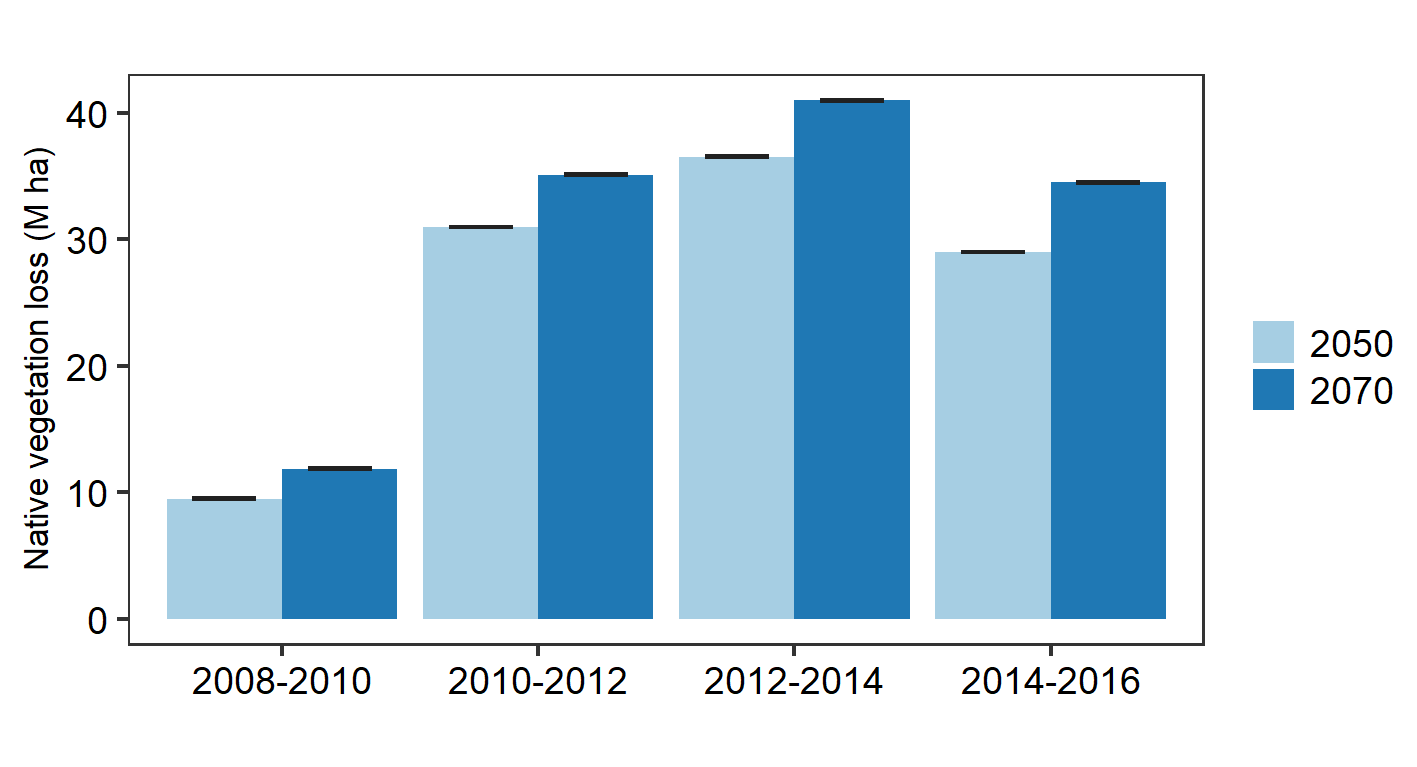
**Figure S4.** Native vegetation loss by 2050 and 2070 in each period analyzed.


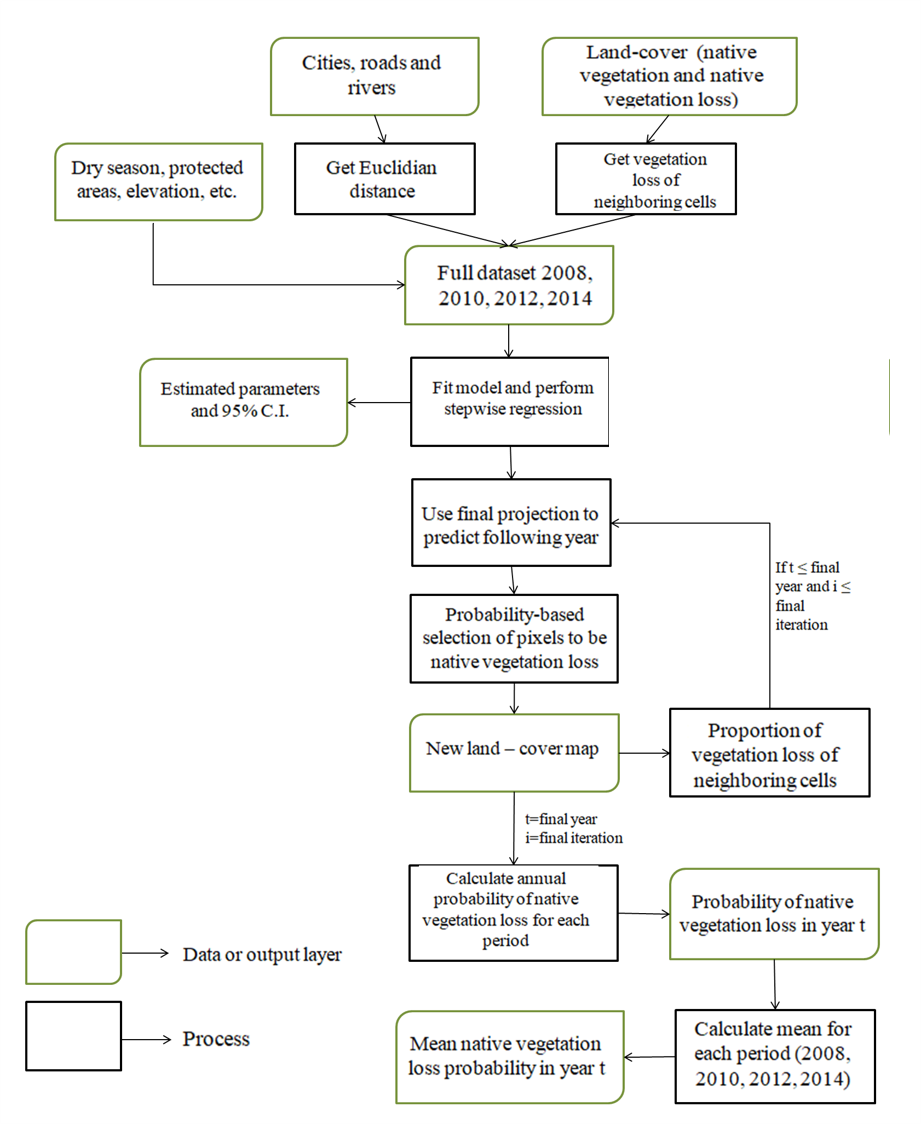


**Figure S5.** Flowchart of modeling procedure ^1^, illustrating the construction and running of the vegetation loss model. *i* is the model iteration, and *t* the time step.

**Table S1**. Input data used to calibrate the model for transition periods 2008-2010, 2010-2012, 2012-2014, and 2014-2016 (dataset name, description, source, reference year and reference).

| **Name** | **Description** | **Source** | **Year** | **Reference** |
| --- | --- | --- | --- | --- |
| Land Cover | Natural (1), Anthropogenic (0) | MapBiomas¹ | 08-10-12-14-16 | 2 |
| Distance to roads | Euclidean distance to nearest road (m) | IBGE² | - | 3 |
| Distance to cities | Euclidean distance to nearest city (m) | IBGE² | - | 4 |
| Dry season length | Number of months with precipitation <100mm | WMO³ | - | 5 |
| Elevation | Altitude (m) | MERIT-DEM^4^ | - | 5 |
| Agricultural potential | Quality of soil/climate for agriculture | IBGE² | - | 5 |
| Distance to Rivers | Euclidean distance to nearest river (m) | IBGE² | - |  |
| Cattle | Change in cattle heads | IBGE² | 08-10, 10-12,  12-14, 14-16 | 6 |
| Permanent Agriculture | Change in permanent agriculture area | IBGE² |  | 6 |
| Annual Crop Agriculture | Change in temporary agriculture area | IBGE² |  | 6 |
| Protected areas | Protected areas (1), unprotected (0) | IBGE² | - | 7 |

¹MapBiomas (<https://mapbiomas.org/download>).

²IBGE – Instituto Brasileiro de Geografia e Estatística (<http://www.ibge.gov.br/home/download/geociencias.shtm>).

³WMO – World Meteorological Organization (http://www.agteca.com/climate.htm).

^4^ MERIT-DEM – Multi-Error-Removed Improved-Terrain DEM (<http://hydro.iis.u-tokyo.ac.jp/~yamadai/MERIT_DEM/>).

**Table S2.** AUC values for each period analyzed.

| **Período** | **2008-2010** | **2010-2012** | **2012-2014** | **2014-2016** |
| --- | --- | --- | --- | --- |
| AUC | 0.78 | 0.83 | 0.85 | 0.84 |

**References**

1 Guerra, A., Roque, F.O., Garcia, L.C., Ochoa-Quintero, J.M., Oliveira, P.T.S., Guariento, R.D., Rosa, I., 2020. Drivers and projections of vegetation loss in the
Pantanal and surrounding ecosystems. Land Use Policy 91, 104388. https://doi.org/10.1016/j.landusepol.2019.104388

2 Brandão Jr, A., Rausch, L., Durán, A.P., Costa Jr., C., Spawn, S.A., Gibbs. L.K. 2020. Estimating the Potential for Conservation and Farming in the Amazon and Cerrado under Four Policy Scenarios. Sustainability 12, 1277. doi:10.3390/su12031277

3 Casella, J., Paranhos-Filho, A.C. 2013. The influence of highway BR262 on the loss of Cerrado vegetation cover in Southwestern Brazil. Oecologia Australis 17, 77-85. http://dx.doi.org/10.4257/oeco.2013.1701.07

4 Seto, K.C., Güneralp, B., Hutyra, L.R., 2012. Global forecasts of urban expansion to 2030 and direct impacts on biodiversity and carbon pools. Proc. Natl. Acad. Sci. 109, 16083–16088. https://doi.org/10.1073/pnas.1211658109

5 Klink, C.A., Machado, R.B. 2005. Conservation of the Brazilian Cerrado. Conservation Biololy 19, 707–713. https://doi.org/10.1111/j.1523-1739.2005.00702.x

6 Lapola, D.M., Martinelli, L.A., Peres, C.A., Ometto, J.P.H.B., Ferreira, M.E., Nobre, C.A., Aguiar, A.P.D., Bustamante, M.M.C., Cardoso, M.F., Costa, M.H., Joly, C.A., Leite, C.C., Moutinho, P., Sampaio, G., Strassburg, B.B.N., Vieira, I.C.G. 2014. Pervasive transition of the Brazilian land-use system. Nature Climate Change 4, 27–35. doi:10.1038/nclimate2056

7 Bensusan, N., 2006. Conservação Da Biodiversidade: Em Áreas Protegidas. Rio de Janeiro:FGV.
